# Supplementary material for: Dynamic fingerprint of fractionalized excitations in single-crystalline Cu3Zn(OH)6FBr
Source: Nat Commun. 2021 May 24;12:3048. doi: 10.1038/s41467-021-23381-9 (PMC8144382; doi:10.1038/s41467-021-23381-9)
Supplement: Supplementary file 1 — Supplementary Information [file 41467_2021_23381_MOESM1_ESM.pdf]

Supplementary Information: Dynamic fingerprint of fractionalized excitations in single-crystalline $Cu_3Zn(OH)_6FBr$

Ying Fu,[1,*] Miao-Ling Lin,[2,3,*] Le Wang,[1] Qiye Liu,[1] Lianglong Huang,[1] Wenrui Jiang,[1] Zhanyang Hao,[1] Cai Liu,[1] Hu Zhang,[4] Xingqiang Shi,[4] Jun Zhang,[2,3,5] Junfeng Dai,[1] Dapeng Yu,[1] Fei Ye,[1,6] Patrick A. Lee,[7] Ping-Heng Tan,[2,3,5,†] and Jia-Wei Mei[1,6,‡]

[1]*Shenzhen Institute for Quantum Science and Engineering, and Department of Physics, Southern University of Science and Technology, Shenzhen 518055, China*
[2]*State Key Laboratory of Superlattices and Microstructures, Institute of Semiconductors, Chinese Academy of Sciences, Beijing 100083, China*
[3]*Center of Materials Science and Optoelectronics Engineering & CAS Center of Excellence in Topological Quantum Computation, University of Chinese Academy of Sciences, 100049 Beijing, China*
[4]*College of Physics Science and Technology, Hebei University, Baoding 071002, China*
[5]*Beijing Academy of Quantum Information Science, Beijing 100193, China*
[6]*Shenzhen Key Laboratory of Advanced Quantum Functional Materials and Devices, Southern University of Science and Technology, Shenzhen 518055, China*
[7]*Department of Physics, Massachusetts Institute of Technology, Cambridge, Massachusetts 02139, USA*
(Dated: April 3, 2021)

CONTENTS

Supplementary Note 1. Estimation of exchange parameters in the kagome compounds 2

Supplementary Note 2. Crystal photograph and thermodynamic characterization 3

Supplementary Note 3. Temperature evolution of the Raman spectra and phonon mode assignment in Cu3Zn 4

Supplementary Note 4. Raman spectra evolution from Cu4 to Cu3Zn and Fano effect in Cu3Zn 5

Supplementary Note 5. Light polarization configurations in angle-resolved polarized Raman Scattering 6

Supplementary Note 6. Second-Harmonic-Generation (SHG) results of Cu3Zn 7

Supplementary Note 7. Raman responses in EuCu3 8

References 8

* These two authors contributed equally.
† phtan@semi.ac.cn
‡ meijw@sustech.edu.cn

Supplementary Note 1. ESTIMATION OF EXCHANGE PARAMETERS IN THE KAGOME COMPOUNDS

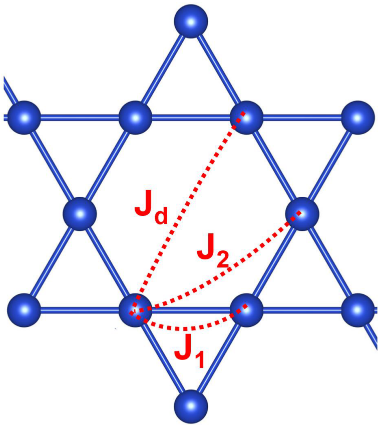

Supplementary Figure 1. Exchange interactions J_1, J_2, and J_d in the Kagome lattice.

We implement the density functional theory (DFT) [1] to estimate the exchange parameters in the kagome compounds. We performed first-principles calculations with the Perdew–Burke–Ernzerhoff revised for solids (PBEsol) functional in generalized gradient approximation (GGA) [2, 3] as implemented in the Vienna Ab Initio Simulation Package (VASP).[4–6] An energy cutoff of 620 eV was used. We used $6 \times 6 \times 4$ Monkhorst-Pack grids [7] for all calculations. All results were obtained with Cu $3d$ valence electrons psudopotential within GGA+U (U_{3d}= 6 eV) scheme.[8]

We fix the lattice constants and relax the atomic positions with a coplanar magnetic structure with negative vector spin chirality in the presence of spin-orbit couplings in our calculations. Exchange interactions can be determined from total energies of various different spin configurations. To determine J_1, J_2, and J_d (see Supplementary Fig. 1), we used the ferromagnetic state, antiferromagnetic state ($\mathbf{q} = 0$), cuboc2 state, and cuboc1 state for a $2 \times 2 \times 1$ supercell as discussed in Fig. 4 of Ref. [9]. Inter-layer couplings J_c are determined by comparing the energies for different stacking patterns of spin configurations. Note for the above interaction terms, we turn off the spin-orbit coupling in our simulations. For the Dzyaloshinski-Moriya (DM) interaction, we turn on spin-orbit couplings and compare the energies of $\mathbf{q} = 0$ antiferromagnetic states with positive and negative vector spin chiralities. The results are listed in Supplementary Table. 1. The nearest neighbor interactions in Cu3Zn and EuCu3 are larger than the experimental values.

Supplementary Table 1. Theoretical results of exchange interaction (in meV) and Cu-O-Cu bonding angle for various materials with the kagome structure. SG denotes the space group. J_c denotes the inter-layer coupling. The references for the lattice constants are also listed.

Formula	SG	J_1 (meV)	J_2 (meV)	J_d (meV)	J_c (meV)	DM (meV)	DM$/J_1$	∠Cu-O-Cu (○)	Reference
$Cu_3Zn(OH)_6FBr$	$P6_3/mmc$	24.13	-0.01	-0.65	1.442	1.12	0.05	117.47	[10]
$YCu_3(OH)_6Cl_3$	$P\bar{3}m1$	10.21	0.12	-0.09	0.040	3.45	0.3	118.60	[11]
$EuCu_3(OH)_6Cl_3$	$P\bar{3}m1$	13.02	0.16	-0.08	0.003	3.83	0.3	120.33	[12]
$SmCu_3(OH)_6Cl_3$	$P\bar{3}m1$	13.55	0.14	-0.08	-0.004	5.91	0.4	120.36	[13]

Supplementary Note 2. CRYSTAL PHOTOGRAPH AND THERMODYNAMIC CHARACTERIZATION

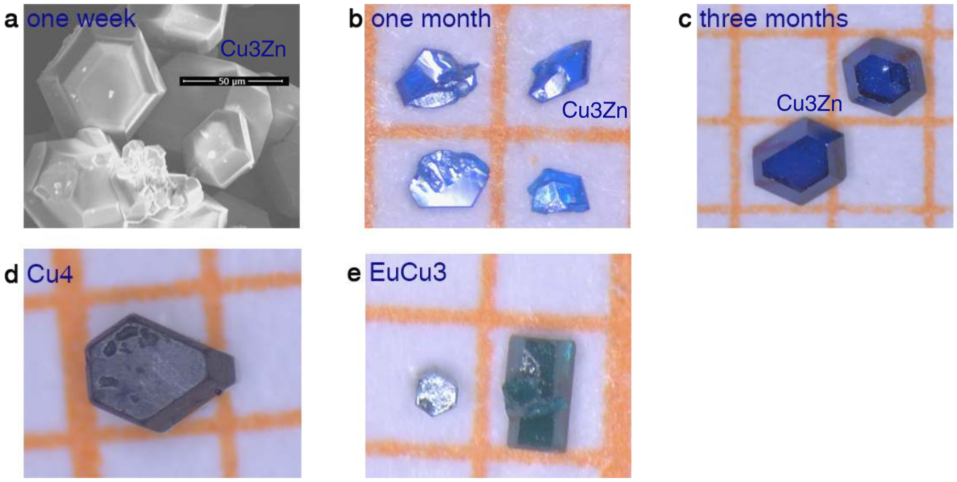

Supplementary Figure 2. Photographs for single crystals of Cu3Zn, Cu4 and EuCu3. Crystal sizes and morphologies of $Cu_3Zn(OH)_6FBr$ (Cu3Zn) for different growth periods: (a) one weak, (b) one month, and (c) three months. (d) $Cu_4(OH)_6FBr$ (Cu4); (e) $EuCu_3(OH)_6Cl_3$ (EuCu3). The yellow grid in (b), (c), (d) and (e) is 1×1 mm^2.

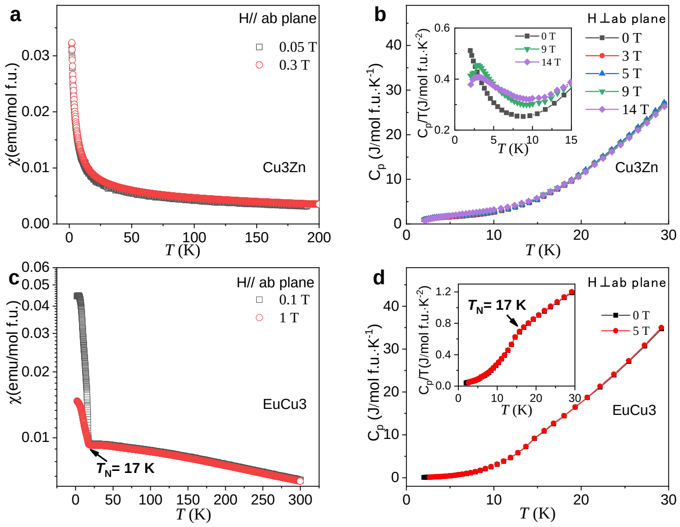

Supplementary Figure 3. Thermodynamic properties of single crystals for Cu3Zn and EuCu3. (a) Temperature dependent magnetic susceptibilities ($\chi = M/H$) at 0.05 T and 0.3 T fields. (b) The temperature dependent specific heat C_p at different magnetic fields in Cu3Zn. The thermodynamic properties of single crystals for Cu3Zn agree well with previous results on the powder samples.[10, 14] (c) The magnetic susceptibilities show the ordering temperature $T_N = 17$ K in EuCu3. The Eu^{3+} with ground state of $^7F_0$ contributes to the Van Vleck paramagnetism (d) The temperature dependent heat capacities C_p in EuCu3.

Supplementary Note 3. TEMPERATURE EVOLUTION OF THE RAMAN SPECTRA AND PHONON MODE ASSIGNMENT IN Cu3Zn

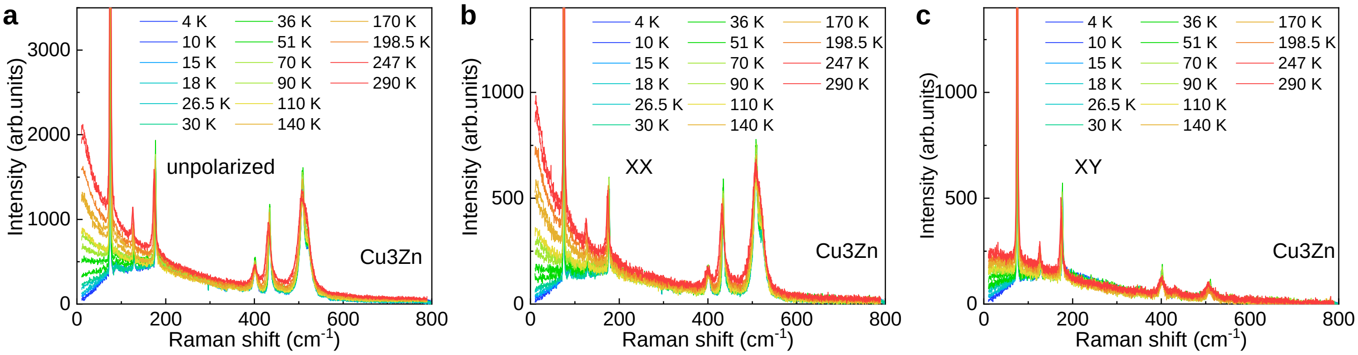

Supplementary Figure 4. Raman spectra in Cu3Zn at different temperatures. (a) Unpolarized Raman spectra in Cu3Zn. (b) Raman spectra in the XX configuration contain the A_{1g} and E_{2g} channel. (c) Raman spectra in the XY configuration contain the E_{2g} channel.

Supplementary Table 2. Phonon mode assignment for Cu3Zn. Cu3Zn crystallizes the space group $P6_3/mmc$ (No. 194) and has Raman-active A_{1g}, E_{1g}, and E_{2g} modes according to the point group representation of D_{6h} ($6/mmm$). E_{1g} is not visible when the light polarization lies in the kagome ab plane, and we have Raman-active phonon modes $\Gamma_{\text{Raman}} = 4A_{1g} + 9E_{2g}$.

Frequency (Exp.) (cm^{-1})	Modes (Exp.)	Frequency (Cal.) (cm^{-1})	Modes (Cal.)	Associated vibrating irons
74.6	E_{2g}	71.2	E_{2g}	Br^-
126.4	E_{2g}	124.11	E_{2g}	Zn^{2+}
172.2	E_{2g}	184.18	E_{2g}	F^-
355.5	E_{2g}	345.37	E_{2g}	O^{2-}
401.5	E_{2g}	396.21	E_{2g}	O^{2-}
430.8	A_{1g}	426.06	A_{1g}	O^{2-}
488.6	E_{2g}, visible in 532 nm	493.47	E_{2g}	O^{2-}
521.1	A_{1g}	508.84	A_{1g}	O^{2-}
920.3	E_{2g}	920.33	E_{2g}	H^+
1016.7	A_{1g}	1082.26	A_{1g}	H^+
1028.2	E_{2g}, weak	1020.11	E_{2g}	H^+
3352.3	?	3333.19	E_{2g}	H^+
3467.5	A_{1g}	3512.14	A_{1g}	H^+

Supplementary Note 4. RAMAN SPECTRA EVOLUTION FROM Cu4 TO Cu3Zn AND FANO EFFECT IN Cu3Zn

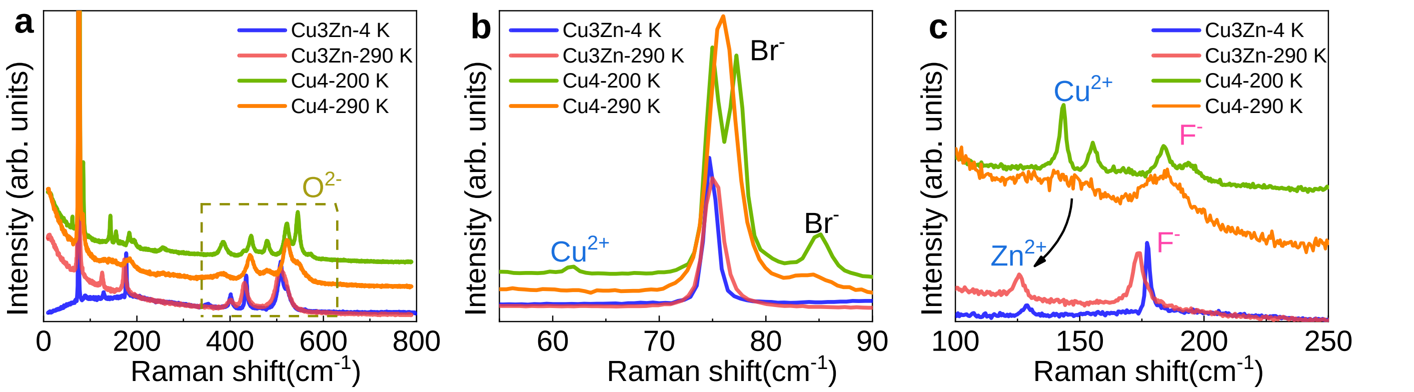

Supplementary Figure 5. Raman spectral evolution from Cu4 to Cu3Zn. (a) Unpolarized Raman spectra for Cu4 and Cu3Zn at selected temperatures. Comparison for phonon modes between 40 cm^{-1} and 90 cm^{-1} in (b), and between 100 cm^{-1} and 250 cm^{-1} in (c) for Cu4 and Cu3Zn. The Cu4 spectra in (a), (b) and (c) have been offset vertically for clarity. The phonon evolution from Cu4 to Cu3Zn displays the difference by substituting the interlayer Cu^{2+} site of Cu4 with Zn^{2+} in Cu3Zn. The parent Barlowite Cu4 transforms to orthorhombic *Pnma* below $T \approx 265$ K, characterized by changes in the relative occupancies of the interlayer Cu^{2+} site. Between 300 cm^{-1} and 600 cm^{-1}, there are several phonon peaks associated with O^{2-} vibrations in Cu4 and Cu3Zn. Cu3Zn displays the in-plane relative vibration of Br^{-} (E_{2g} mode) at 75 cm^{-1}, and has no Raman-active mode related to the kagome Cu^{2+} vibrations since Cu^{2+} is the inversion center. The Br^{-} phonon mode splits into two peaks in Cu4 due to the superlattice folding in the orthorhombic Pnma phase at low temperature. An additional Br^{-} peak at 85 cm^{-1} appears in Cu4, related to the Br vibrations along the c-axis. The kagome layers in Cu4 are distorted at low temperature, signaled by a new phonon mode for the kagome Cu^{2+} vibration at 62 cm^{-1}. Cu3Zn displays sharp E_{2g} modes at 125 cm^{-1} and 173 cm^{-1} corresponding to in-plane relative movements for Zn^{2+} and F^{-}, respectively. The corresponding modes (interlayer Cu^{2+} and F^{-} vibrations) in Cu4 are broad at 290 K due to the randomly distributed interlayer Cu^{2+} and split into two peaks at 200 K.

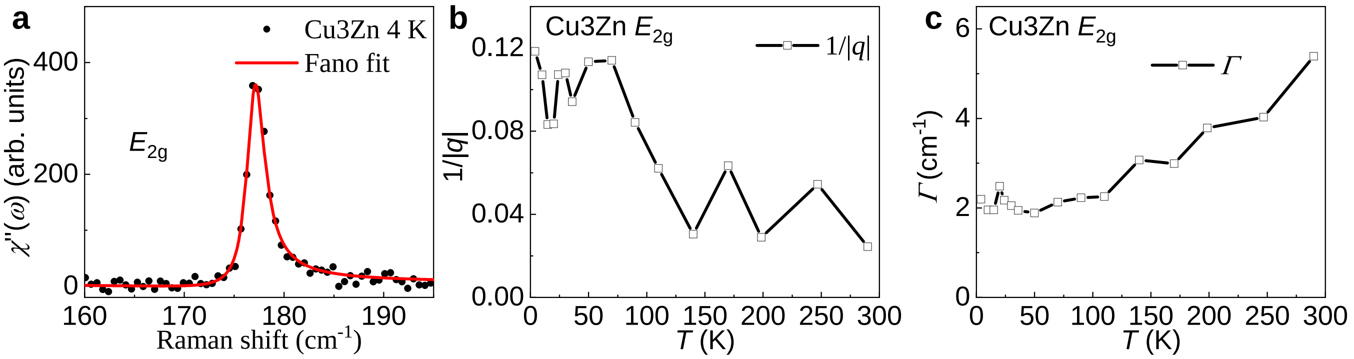

Supplementary Figure 6. Fano lineshape of the E_{2g} F^{-} phonon peak at 173 cm^{-1} in Cu3Zn. (a) Fano lineshape for the E_{2g} in-plane phonon mode related to F atomic movement. Temperature dependent Fano asymmetric parameter $1/|q|$ in (b) and the width Γ in (c). The asymmetric Fano lineshape provides an additional probe of the magnetic degree of freedom.

Supplementary Note 5. LIGHT POLARIZATION CONFIGURATIONS IN ANGLE-RESOLVED POLARIZED RAMAN SCATTERING

The polarized Raman measurements with light polarized in the ab kagome plane of samples were performed in parallel (XX), perpendicular (XY), and X-only polarization configurations. Two typical polarization configurations were utilized to measure the angle-resolved polarized Raman (ARPR) spectra: i) a half-wave plate was put after the polarizer in the incident path to vary the angles between the polarization of incident laser and the analyzer with the fixed vertical polarization, which can be denoted as the X-only configuration; ii) a half-wave plate is allocated in the common path of the incident and scattered light to simultaneously vary their polarization directions, while the polarizations of incident laser and analyzer were parallel or perpendicular to each other. By rotating the fast axis of the half-wave plate with an angle of $\theta/2$, the polarization of incident and/or scattered light is rotated by θ.

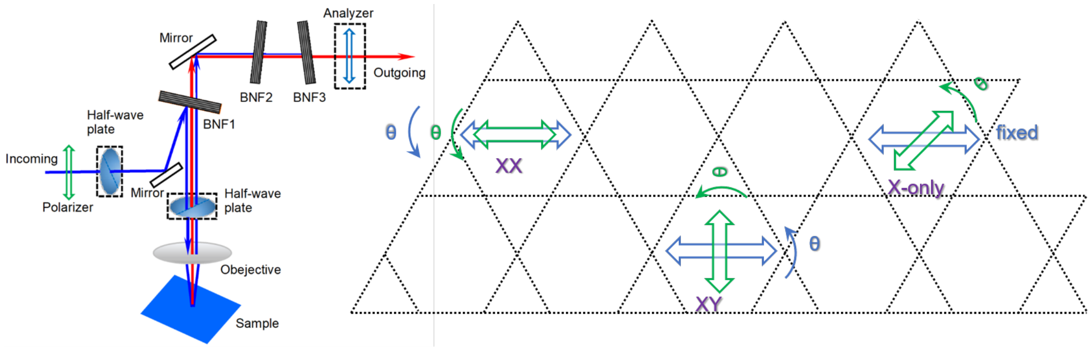

Supplementary Figure 7. Three polarization configurations in the ARPR response. In the XX (XY) configuration, the incoming and outgoing light polarizations are parallel (perpendicular) and we rotate both of them simultaneously. In the X-only configuration, the outgoing light polarization is fixed and we rotate the incoming light polarization only.

Supplementary Note 6. SECOND-HARMONIC-GENERATION (SHG) RESULTS OF Cu3Zn

SHG measurements were performed using a homemade confocal microscope in a back-scattering geometry. A fundamental wave centered at 800 nm was used as excitation source, which was generated from a Ti-sapphire oscillator (Chameleon Ultra II) with an 80 MHz repetition frequency and a 150 fs pulse width. After passing through a 50× objective, the pump beam was focused on the sample with a diameter of 2 μm. The scattering SHG signals at 400 nm were collected by the same objective and led to the entrance slit of a spectrometer equipped with a thermoelectrically cooled CCD. Two shortpass filters were employed to cut the fundamental wave.

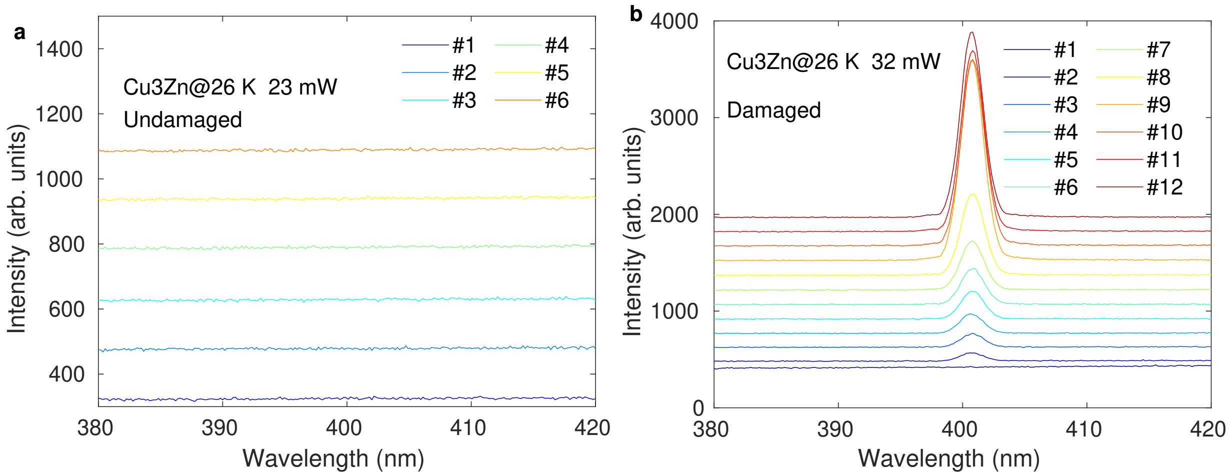

Supplementary Figure 8. SHG in Cu3Zn at 26 K with different laser powers. (a) SHG measurements in the same spot of sample taken every 5 seconds (from #1 to #6). At 23 mW, SHG signals in Cu3Zn sample are absent, implying that inversion symmetry remains preserved. (b) A series of SHG measurements under the excitation power of 32 mW in the same point of the sample taken every 5 seconds (from #1 to #12). A remarkable SHG signal at 400 nm is detectable after a 10-second exposure, which dramatically enhances as the time increases. Due to the damage or degradation of Cu3Zn under high power excitation, the inversion symmetry breaking induces a strong SHG signals in sample. By comparison, we conclude that undamaged Cu3Zn single crystal presents spatial inversion symmetry at low temperature. The lines have been offset vertically for clarity.

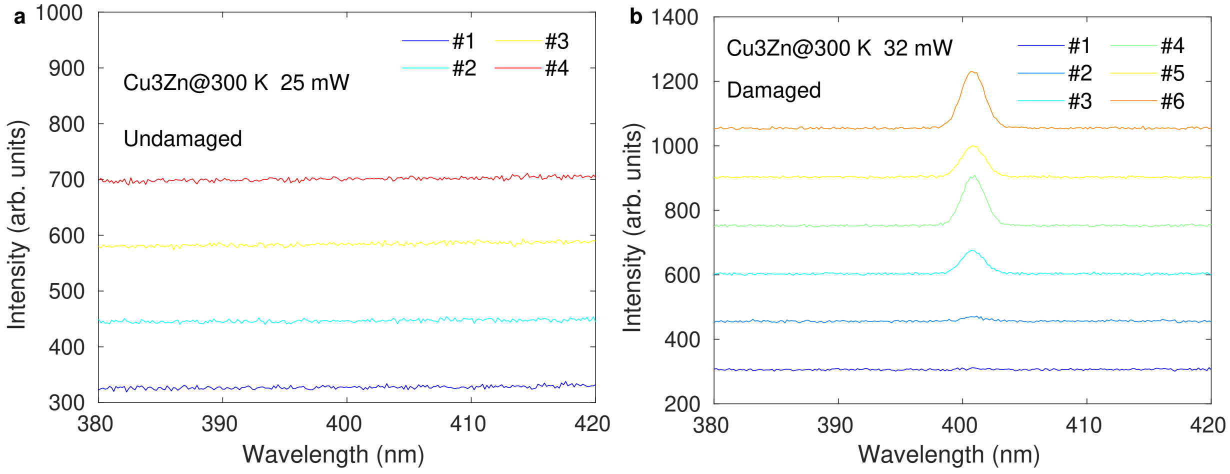

Supplementary Figure 9. SHG in Cu3Zn at 300 K with different laser powers. (a) and (b) represent the successive SHG measurements in the same point of sample taken every 5 seconds with excitation powers at 25 mW and 32 mW, respectively. There are no SHG signals at the excitation power of 25 mW, whereas strong SHG signals appear at the excitation power above 32 mW after a 10-second exposure. By comparison, damage or degradation in crystal structure under high power excitation induces a detectable SHG signal, implying that inversion symmetry presents in undamaged Cu3Zn at room temperature. The lines have been offset vertically for clarity.

Supplementary Note 7. RAMAN RESPONSES IN EuCu3

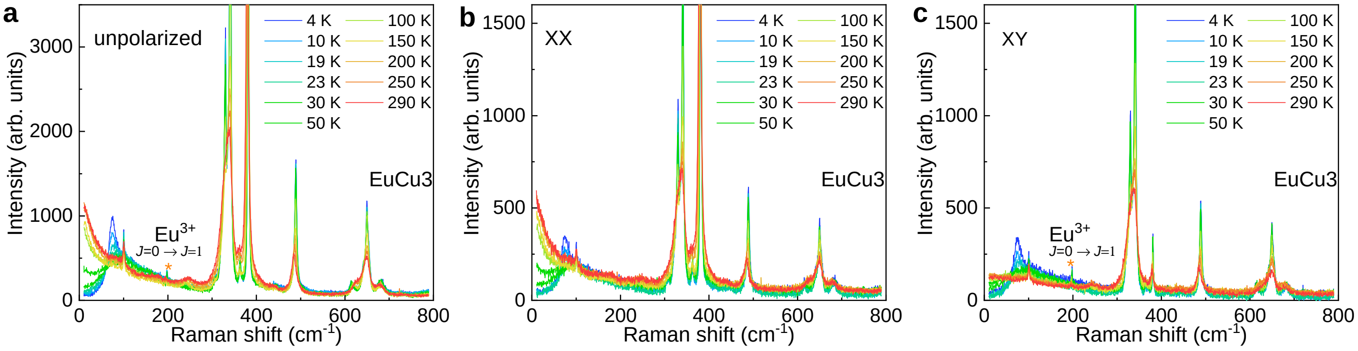

Supplementary Figure 10. Raman spectra of EuCu3 at different temperatures. (a) Unpolarized Raman spectra. (b) Raman spectra in the XX configuration in EuCu3 contain the A_g and E_g channel. (c) Raman spectra in the XY configuration contain the E_g and A_{2g} channel. For Eu^{3+}, we observe the A_{2g} excitation of the $4f^6$ configuration with the transition from $^7F_{J=0}$ to $^7F_{J=1}$.

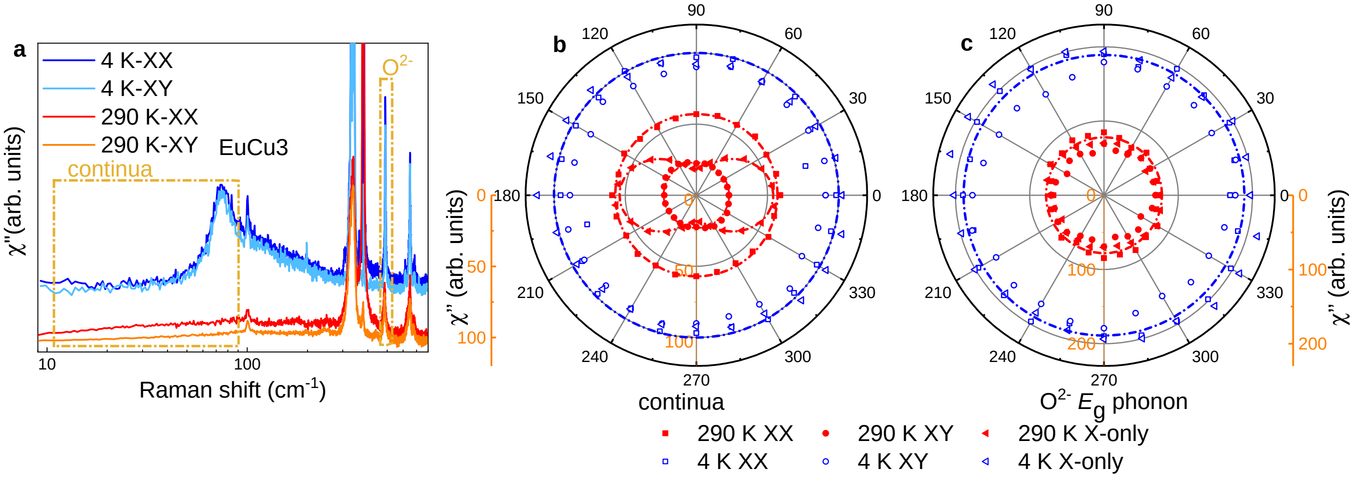

Supplementary Figure 11. Rotation symmetry of Raman dynamics for lattice vibrations and magnetic excitations in EuCu3. We monitor the selected magnetic continuum at low frequency and the O^{2-} E_g mode in (a). (b) ARPR dependence of the integrated Raman continuum from 9 to 80 cm^{-1}. The continua at 290 K follow the $\cos^2(\theta)$ function for the A_{1g} channel, while in other cases, the continua remain constant. (c) ARPR dependence of the O^{2-} E_g phonon (487 cm^{-1}) scattering intensity. Its Raman intensity is independent of θ.

[1] R. O. Jones, Density functional theory: Its origins, rise to prominence, and future, Rev. Mod. Phys. **87**, 897 (2015).

[2] J. P. Perdew, K. Burke, and M. Ernzerhof, Generalized gradient approximation made simple, Phys. Rev. Lett. **77**, 3865 (1996).

[3] J. P. Perdew, A. Ruzsinszky, G. I. Csonka, O. A. Vydrov, G. E. Scuseria, L. A. Constantin, X. Zhou, and K. Burke, Restoring the density-gradient expansion for exchange in solids and surfaces, Phys. Rev. Lett. **100**, 136406 (2008).

[4] G. Kresse and J. Furthmüller, Efficient iterative schemes for ab initio total-energy calculations using a plane-wave basis set, Phys. Rev. B **54**, 11169 (1996).

[5] M. Gajdoš, K. Hummer, G. Kresse, J. Furthmüller, and F. Bechstedt, Linear optical properties in the projector-augmented wave methodology, Phys. Rev. B **73**, 045112 (2006).

[6] J. Hafner, Ab-initio simulations of materials using vasp: Density-functional theory and beyond, J. Comput. Chem. **29**, 2044 (2008).

[7] H. J. Monkhorst and J. D. Pack, Special points for brillouin-zone integrations, Phys. Rev. B **13**, 5188 (1976).

[8] A. I. Liechtenstein, V. V. Anisimov, and J. Zaanen, Density-functional theory and strong interactions: Orbital ordering in mott-hubbard insulators, Phys. Rev. B **52**, R5467 (1995).

[9] L. Messio, C. Lhuillier, and G. Misguich, Lattice symmetries and regular magnetic orders in classical frustrated antiferromagnets, Phys. Rev. B **83**, 184401 (2011).

[10] Z. Feng, Z. Li, X. Meng, W. Yi, Y. Wei, J. Zhang, Y.-C. Wang, W. Jiang, Z. Liu, S. Li, F. Liu, J. Luo, S. Li, G. qing Zheng, Z. Y. Meng, J.-W. Mei, and Y. Shi, Gapped Spin-1/2 Spinon Excitations in a New Kagome Quantum Spin Liquid Compound $Cu_3Zn(OH)_6FBr$, Chinese Physics Letters **34**, 077502 (2017).

[11] A. Zorko, M. Pregelj, M. Gomilšek, M. Klanjšek, O. Zaharko, W. Sun, and J.-X. Mi, Negative-vector-chirality 120° spin structure in the defect- and distortion-free quantum kagome antiferromagnet $YCu_3(OH)_6Cl_3$, Phys. Rev. B **100**, 144420 (2019).

[12] P. Puphal, K. M. Zoch, J. Désor, M. Bolte, and C. Krellner, Kagome quantum spin systems in the atacamite family, Phys. Rev. Materials **2**, 063402 (2018).

[13] W. Sun, Y. X. Huang, Y. Pan, and J. X. Mi, Strong spin frustration and negative magnetization in $LnCu_3(OH)_6Cl_3$ (Ln = Nd and Sm) with triangular lattices: the effects of lanthanides, Dalton Trans. **46**, 9535 (2017).

[14] Y. Wei, Z. Feng, W. Lohstroh, C. dela Cruz, W. Yi, Z. F. Ding, J. Zhang, C. Tan, L. Shu, Y.-C. Wang, J. Luo, J.-W. Mei, Z. Y. Meng, Y. Shi, and S. Li, Evidence for a Z_2 topological ordered quantum spin liquid in a kagome-lattice antiferromagnet (2017), arXiv:1710.02991.
